# Rifaximin Alters Intestinal Microbiota and Prevents Progression of Ankylosing Spondylitis in Mice

**DOI:** 10.3389/fcimb.2019.00044

**Published:** 2019-03-04

**Authors:** Lianjun Yang, Bin Liu, Junchi Zheng, Jincheng Huang, Qinghao Zhao, Jinshi Liu, Zhihai Su, Min Wang, Zhifei Cui, Tingxuan Wang, Weicong Zhang, Qingchu Li, Hai Lu

**Affiliations:** ^1^Department of Orthopedic Surgery, Orthopaedic Hospital of Guangdong Province, Academy of Orthopedics of Guangdong Province, The Third Affiliated Hospital, Southern Medical University, Guangzhou, China; ^2^Department of Orthopedics, Zhengzhou University People's Hospital, Henan Provincial People's Hospital, Zhengzhou, China

**Keywords:** ankylosing spondylitis, rifaximin, gut microbiota, inflammatory response, intestinal epithelial barrier

## Abstract

Recently, accumulating evidence has suggested that gut microbiota may be involved in the occurrence and development of ankylosing spondylitis (AS). It has been suggested that rifaximin have the ability to modulate the gut bacterial communities, prevent inflammatory response, and modulate gut barrier function. The goal of this work is to evaluate the protective effects of rifaximin in fighting AS and to elucidate the potential underlying mechanism. Rifaximin were administered to the proteoglycan (PG)-induced AS mice for 4 consecutive weeks. The disease severity was measured with the clinical and histological of arthritis and spondylitis. Intestinal histopathological, pro-inflammatory cytokine levels and the intestinal mucosal barrier were evaluated. Then, western blot was performed to explore the toll-like receptor 4 (TLR-4) signal transducer and NF-κB expression. Stool samples were collected to analyze the differences in the gut microbiota via next-generation sequencing of 16S rDNA. We found that rifaximin significantly reduced the severity of AS and resulted in down-regulation of inflammatory factors, such as TNF-α, IL-6, IL-17A, and IL-23. Meanwhile, rifaximin prevented ileum histological alterations, restored intestinal barrier function and inhibited TLR-4/NF-κB signaling pathway activation. Rifaximin also changed the gut microbiota composition with increased *Bacteroidetes/Firmicutes* phylum ratio, as well as selectively promoting some probiotic populations, including *Lactobacillales*. Our results suggest that rifaximin suppressed progression of AS and regulated gut microbiota in AS mice. Rifaximin might be useful as a novel treatment for AS.

## Introduction

Ankylosing Spondylitis (AS) is a chronic systemic autoinflammatory disease mainly affects sacroiliac joints and axial skeleton. To date, a lack of knowledge regarding the precise molecular mechanisms underlying AS progression limits the ability to halt the progression of this disease. Although human leukocyte antigen B27 (HLA-B27) is estimated to be linked to 90–95% of AS cases (Martín-Esteban et al., 2017), the relationship between HLA-B27 and AS has not been fully elucidated. The cause of AS cannot be fully explained by a genetic predisposition alone, explaining the ongoing need to identify additional predisposing factors. The consensus is that AS is caused by coaction of heredity and environmental factors (Yang et al., 2016).

Changes in the commensal intestinal microbiota has been associated with several autoimmune diseases, including multiple sclerosis (Mestre et al., 2018), inflammatory bowel disease (IBD) (Sheehan et al., 2015), and type 1 diabetes (Meijnikman et al., 2018). Many studies have shown that AS and IBD share similar genetic risk factors and etiopathogeneses (Rashid et al., 2013). Studies based on next-generation sequencing technology, and bioinformatics suggest a significant relationship between the pathogenesis of AS and dysbiosis of intestinal microbiota. Wen et al. (2017) demonstrated that intestinal microflora in AS patients were enriched in *Prevotella melaninogenica, Prevotella copri*, and *Prevotella* sp. C561, along with a lower level of *Bacteroides* spp. In a similar study, in the terminal ileum, AS patients had significantly increased levels of five bacterial families and reduced levels of two bacterial families, when compared to healthy controls (Costello et al., 2014). In animal experiment, HLA-B27 transgenic rats housed in a germ-free environment do not appear features of spondyloarthritis (SpAs). However, HLA-B27 transgenic rats when transferred to a conventional rat colony, these rats appeared AS-like symptoms including colitis and arthritis (Rath et al., 1996). This suggests that intestinal flora takes a critical role in the pathogenesis of AS, and gut microbiota may therefore become a potential target for AS treatment.

Using antibiotics to manipulate gut microbiota may represent a potentially effective treatment option for AS. Previous research has shown that AS patients treated with moxifloxacin, a fourth-generation fluoroquinolone, presented with significantly reduced inflammatory symptoms (Ogrendik, 2007). Rifaximin is a gastrointestinal selective antibiotic that is almost no absorption into the blood with less adverse reaction (Ponziani et al., 2017). Rifaximin acts by inhibiting bacterial DNA-dependent RNA polymerase. While the full effect of rifaximin on gut flora remains limited. Previous studies have shown that rifaximin can alter intestinal flora, inhibit bacterial attachment, prevent intestinal inflammation, and modulate gut barrier function (Xu et al., 2014; Lopetuso et al., 2018). This special feature distinguishes rifaximin from other systemic antibiotics. Most studies up to now have focused on evaluating the effect of rifaximin on gut-liver axis or gut-brain axis (Ponziani, 2015; Wang et al., 2018). However, it is not clear whether orally administered rifaximin can prevent the development of AS by down-regulation of the inflammatory response triggered by gut microbes. In this research, we have evaluated the effects of rifaximin on the severity of AS, inflammatory response, and the changes in intestinal flora. To our knowledge, it is the first study to evaluate the effect of rifaximin on the gut-bone axis in AS.

## Materials and Methods

### Construction of an AS Mouse Model and Drug Administration

Female BALB/c mice, retired breeders aged 8 months, were obtained from Beijing HFK Bioscience Co. Ltd, China. They were housed in an air-conditioned room with the laboratory temperature at 22–24°C with 12 h light–dark cycle. This study was carried out in accordance with the recommendations of the Care and Use of Laboratory Animals (Ministry of Health, China). The protocol was approved by the Animal Experiment Committee of the Southern Medical University. The proteoglycan (PG)-induced AS model has been described previously (Boldizsar et al., 2009; Ishikawa et al., 2014). Mice were immunized, via intraperitoneal injection with an emulsion of 100 μg of cartilage PGs (Sigma, St. Louis, MO, USA), and 2 mg dimethyldioctadecylammonium adjuvant (Sigma, St. Louis, MO, USA) on day 0, 21, and 42.

**Figure 1:**
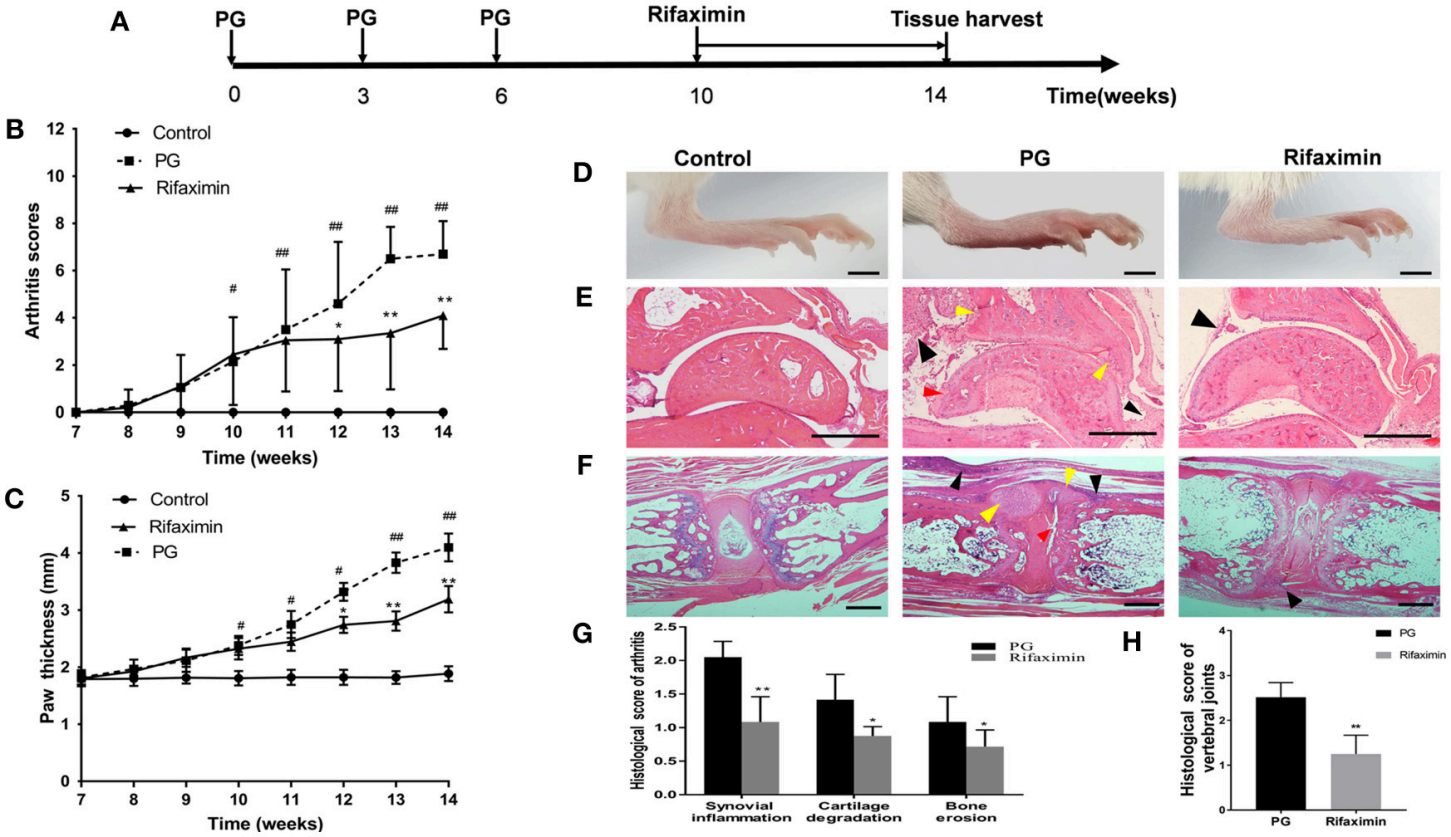
Rifaximin attenuates the progression and severity of AS mice. **(A)** Schematic representation of study design. Arthritis scores **(B)** and hind paw thickness **(C)** from the three groups (*n* = 10 mice per group). **(D)** Representative photographs of hind paws from the different groups (Scale bar is 2 mm). **(E)** Representative pictures of sectioned ankles. PG group was characterized by synovial inflammation (black arrow head), narrowing of joint space, cartilage degradation (red arrow head) and bone erosion (yellow arrow head) (H&E staining, Scale bar is 500 μm). **(F)** Representative pictures of vertebral joints. PG group was characterized by severe inflammatory cells accumulate at the periphery of the disc (black arrow head), excess bone matrix formation (yellow arrow head), and part of IVD destruction (red arrow head) (Scale bar is 50 μ m). **(G)** Histologic analysis of paw joint sections from PG or rifaximin mice (*n* = 6 mice per group). **(H)** Histologic analysis of vertebral joint sections from PG or rifaximin mice (*n* = 6 mice per group). Values are presented as means ± SD (^#^*P* < 0.05 and ^##^*P* < 0.01 vs. control; ^*^P < 0.05 and ^**^P < 0.01 vs. PG alone).

The mice were assigned randomly to three treatment groups (*n* = 10 per group): (1) a control group containing healthy mice; (2) an AS model group (PG group), in which AS mice were treated with normal saline; (3) a rifaximin group in which PG mice were treated with rifaximin (ALFA WASSERMANN S.p.A, Italy) by oral gavage (100 mg/kg/day) for 4 weeks. Mice started treatment 10 weeks after PG induction, and for 4 weeks thereafter. The rifaximin dosage was chosen based on previously published data (Kang et al., 2016). The experiment lasted a total of 14 weeks (Figure 1A).

### Clinical and Histological Assessments of Arthritis and Spondylitis

The severity of arthritis was evaluated every day as a previous publication (Ishikawa et al., 2014). The following clinical score measurement was used: 0 = normal; 1 = focal slight swelling and/or redness in one digit; 2 = moderate redness and moderate paw swelling and one or more joints; 3 = marked swelling and erythema in the paw, and all joints, and ankles; 4 = swelling of the entire paw. Accumulated scores for all four paws of each mouse (maximum score of 16) were used to determine disease severity and progression. The maximal thickness observed in either of the 2 hind paws of each mouse was also measured at the same time by Vernier caliper. Upon sacrifice, hind paws and spine were dissected then fixed in 4% phosphate-buffered paraformaldehyde, and further decalcified in 10% Titriplex EDTA for 1 month. The decalcified tissues were embedded in paraffin, and sectioned 5 μm and stained with hematoxylin and eosin. The histological arthritis score was determined in a blinded manner for changes in synovial inflammation, cartilage degradation, and bone erosion as previously described(Ishikawa et al., 2016; Hablot et al., 2017). Synovial inflammation was scored as following scale: 0 = normal, 1 = slight thickening of the lining layer with some infiltrating cells in the sublining layer, 2 = moderate thickening of the lining layer with a moderate number of infiltrating cells in the sublining layer, 3 = severe inflammation with a massive immune cell infiltrate into the synovium. Cartilage degradation was scored as following scale: 0 = normal, 1 = mild cartilage destruction, 2 = evidence of cartilage destruction with synovial cells invasion, 3 = severe loss of cartilage. Bone erosion was scored as following scale: 0 = normal, 1 = mild loss of cortical bone at few sites, 2 = moderate loss of cortical and trabecular bone and 3 = marked loss of bone at many sites.

Histological score of the vertebral joint was determined on a scale of 1–4 (Haynes et al., 2012): 1 = few inflammatory cell accumulation around the intervertebral disc (IVD) and/or infiltration of the annulus fibrosus; 2 = mild inflammation, absorption/erosion of IVD (< 50% of IVD); 3 = severe inflammatory, essentially complete resorption (>50%) of the IVD; 4 = cartilaginous/bony ankylosis. An overall average score for each mouse was then generated by averaging the scores for ten vertebral joints.

### ELISA Assay

At the end of week14, the mice were anesthetized with avertin and blood was collected by heart puncture. After coagulation and serum samples were separated by centrifugation at 1,500 × g for ten min and stored in the freezer at −80°C. TNF-α, IL-6, IL-17A, and IL-23 levels were measured by a commercial ELISA kit (ABclonal Technology TM, Wuhan, China), following the manufacturer's instructions.

### Intestinal Morphology and Immunohistochemistry

After sacrificed, small intestine tissue was quickly removed and fixed in 4% phosphate-buffered paraformaldehyde and processed by standard procedures for paraffin embedding. Standard sections of 5 μm were stained by means of hematoxylin eosin (HE) (Xue et al., 2018), and used for immunohistochemical analyses. Briefly, slides were deparaffinized, rehydrated through graded alcohol and washed in PBS, immersed in 0.3% hydrogen peroxide for 15 min, and then underwent antigen retrieval, washed in PBS, and the sections were immersed in goat serum for 20 min to block non-specific binding. After blocking, the sections were incubated with rabbit anti ZO-1 (diluted 1:150; Thermo Fisher Scientific, Carlsbad, USA), and occludin antibodies (diluted 1:200; Thermo Fisher Scientific, Carlsbad, USA) (Ruan et al., 2014). Slides were photographed and analyzed under the Zeiss microscope (Carl Zeiss, New York, USA). Villus height was measured from the tip of the villus to the villus-crypt junction. Crypt depth was defined as the bottom of the villus to the lamina propria. The villus height and crypt depth of 9 intact, well-oriented villi were measured per section (Lei et al., 2015). The optical density of the abundance of ZO-1 and occludin proteins examined using Image-Pro Plus version 6.0 software. The arithmetic mean of the quantification in five fields of each section was determined.

### *In vivo* Gut Permeability Test

Briefly, mice were fasted without water supplement for 6 h, and then treated with fluorescein isothiocyanate conjugated with 4 kDa dextran (DX-4000-FITC, Sigma-Aldrich) by gavage (600 mg/kg body weight, 125 mg/ml), as previously described (Cani et al., 2008; Hwang et al., 2015). 1 h later, the blood samples (300 μl) were collected from behind the eye sockets, and the serum prepared for fluorescence measurements. Serum was diluted with an equal volume of PBS and analyzed for FITC concentration with a fluorescence spectrophotometer at an excitation wavelength of 485 nm and emission wavelength of 535 nm. Standard curves of DX-4000-FITC concentrations were obtained by diluting DX-4000-FITC solution with PBS.

### 16S rDNA High Throughput Sequencing and Data Processing

Stool samples were collected into sterile containers 12 h after the last gavage and immediately frozen at −80°C. Microbial DNA was extracted from the fecal material of each sample using the QIAamp DNA Stool Minikit (Qiagen Ltd, Strasse, Germany). The next-generation sequencing of 16S rDNA using the Illumina HiSeq PE250 was performed by Realbio Genomics Institute (Shanghai, China). V3–V4 region of the 16S rDNA gene sequence were amplified from genomic DNA using the primer pair, F341 (5′-ACTCCTACGGGRSGCAGCAG-3′) and R806 (5′- GGACTACVVGGGTATCTAATC-3′). The raw data were then subjected to a quality control procedure using UPARSE. Cluster reads into operational taxonomic units (OTUs) of ≥97% similarity using Usearch. Principal coordinate analysis (PCoA), heatmap analysis, and species abundance analysis were performed using R. Relative abundance of gut microbiota was compared between the control, PG, and rifaximin groups using linear discriminant analysis effect size (LEfSe) coupled with the Kruskal-Wallis rank sum test. Thresholds for the LEfSe analysis was log linear discriminant analysis (LDA) score >2. PICRUS was used to predict metagenome function from the 16S rRNA data (Qi et al., 2018). The 16S rRNA gene sequences have been deposited into the NCBI Sequence Read Archive (SRA) database under accession number SRP182773.

### Western Blot Analysis

Ileal tissue obtained from the three groups was frozen in liquid nitrogen, homogenized in RIPA buffer, and then centrifuged for 20 min. Nuclear and cytosolic protein was isolated from the tissue using a nuclear and cytoplasmic protein extraction kit (Beyotime, Beijing, China). The BCA protein assay kit (Beyotime, Beijing, China) was used to determined protein concentrations. After this, equal amounts of protein were electrophoresed in gradient SDS-PAGE gels, and then electrotransferred onto a PVDF membrane (Millipore) using a semidry transfer system (Bio-Rad). After blocking with 5% bovine serum albumin (BSA) for 1 h at room temperature, the membranes were incubated with specific primary antibodies overnight at 4°C. The sources of antibodies and dilutions used were as follows: anti-ZO-1 (1:500; Thermo Fisher Scientific, Carlsbad, USA), anti-occludin (1:500;Thermo Fisher Scientific, Carlsbad, USA), anti-toll-like receptor 4 (TLR-4, 1:300; Abcam, Cambridge, UK), anti-NF-κB p65 (1:500 Thermo Fisher Scientific, Carlsbad, USA), anti-β-Actin (1:5000; Bioss, Beijing, China), and anti-Lamin A (1:1000 Thermo Fisher Scientific, Carlsbad, USA). Following this, the membrane was incubated with horseradish peroxidase (HRP)-conjugated secondary antibody for 1 h at room temperature, and detected by using BeyoECL Plus (Beyotime, Beijing, China), and visualized using the Gene-Gnome Bio Imaging System (Syngene Bio-imaging, Frederick, MD, USA).

### Statistical Analysis

Quantitative data are expressed as means ± standard deviation (S.D). SPSS 22.0 software was employed to analyze all data. Statistical analysis was carried out using one-way analysis of variance (ANOVA), followed by Bonferroni's multiple comparison test. A *p* < 0.05 indicated statistically significant differences.

## Results

### Rifaximin Attenuates the Progression and Severity of AS Mice

To determine the effectiveness of rifaximin treatment on arthritis, we compared arthritis levels from the three experimental groups in mice. Mice from the control group exhibited no adverse effects, whereas those from the PG group showed developed red swelling in the foot and toes. However, mice treatment with 100 mg/kg of rifaximin, the red swelling was alleviated. Arthritis scores and foot paw thickness in rifaximin-treated mice were significantly lower than in PG mice (Figures 1B,C). Figure 1D shown representative photographs of the hind paws from the different groups.

H&E staining showed that histological features of the ankles from the control group exhibited no sign of inflammation, or cartilage degradation, and a clear synovial space. However, the ankles of mice from the PG group were arthritic, and characterized by synovial inflammation, cartilage degradation, and bone erosion (Figure 1E). Meanwhile, we observed that vertebral joints were destructed in PG mice (Figure 1F). There was a broad range of inflammatory cells accumulate at the periphery of the disc with excess bone matrix formation and IVD destruction. These histological features of ankles and vertebral joints were less pronounced in the rifaximin treated mice. Histological scores of the peripheral arthritis and vertebral joints were significantly decreased in rifaximin treated mice (Figures 1G,H). Taken together, these finding indicate that rifaximin inhibits the development and severity of AS in mice.

### Rifaximin Reduces the Expression of Inflammatory Cytokines

**Figure 2:**
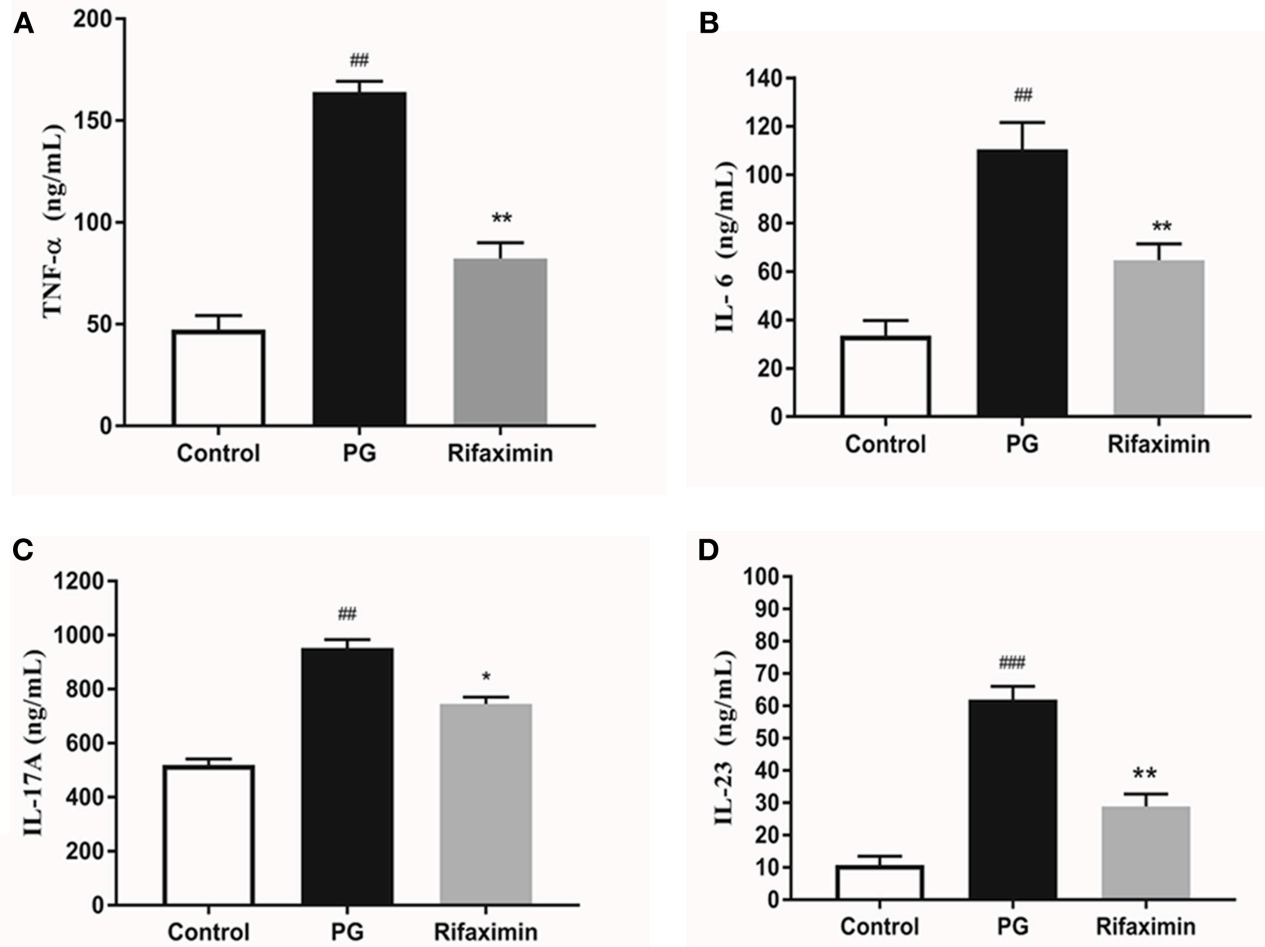
Rifaximin reduces the expression of inflammatory cytokines in AS mice. Mice were orally administered with rifaximin (100 mg/kg/day) for 4 weeks. The serum level of TNF-α, IL-6, IL-17A, and IL-23 were determined by ELISA. Data are expressed as means ± standard deviation from 10 mice per group. **(A)** TNF-α; **(B)** IL-6; **(C)** IL-17A; **(D)** IL-23 (^##^*P* < 0.01, and ^###^*P* < 0.001 vs. control; ^*^P < 0.05, and ^**^P < 0.01 vs. PG alone).

As demonstrated in Figure 2, the serum level of TNF-α, IL-6, IL-17A, and IL-23 in the PG group were significantly increased as compared with the control group. However, mice treatment with rifaximin, the TNF-α, IL-6, IL-17A, and IL-23 were significantly reduced compared to the PG group. These findings indicate that rifaximin can decrease the levels of pro-inflammatory factors.

### Rifaximin Reduces Ileum Histological Alterations in AS Mice

**Figure 3:**
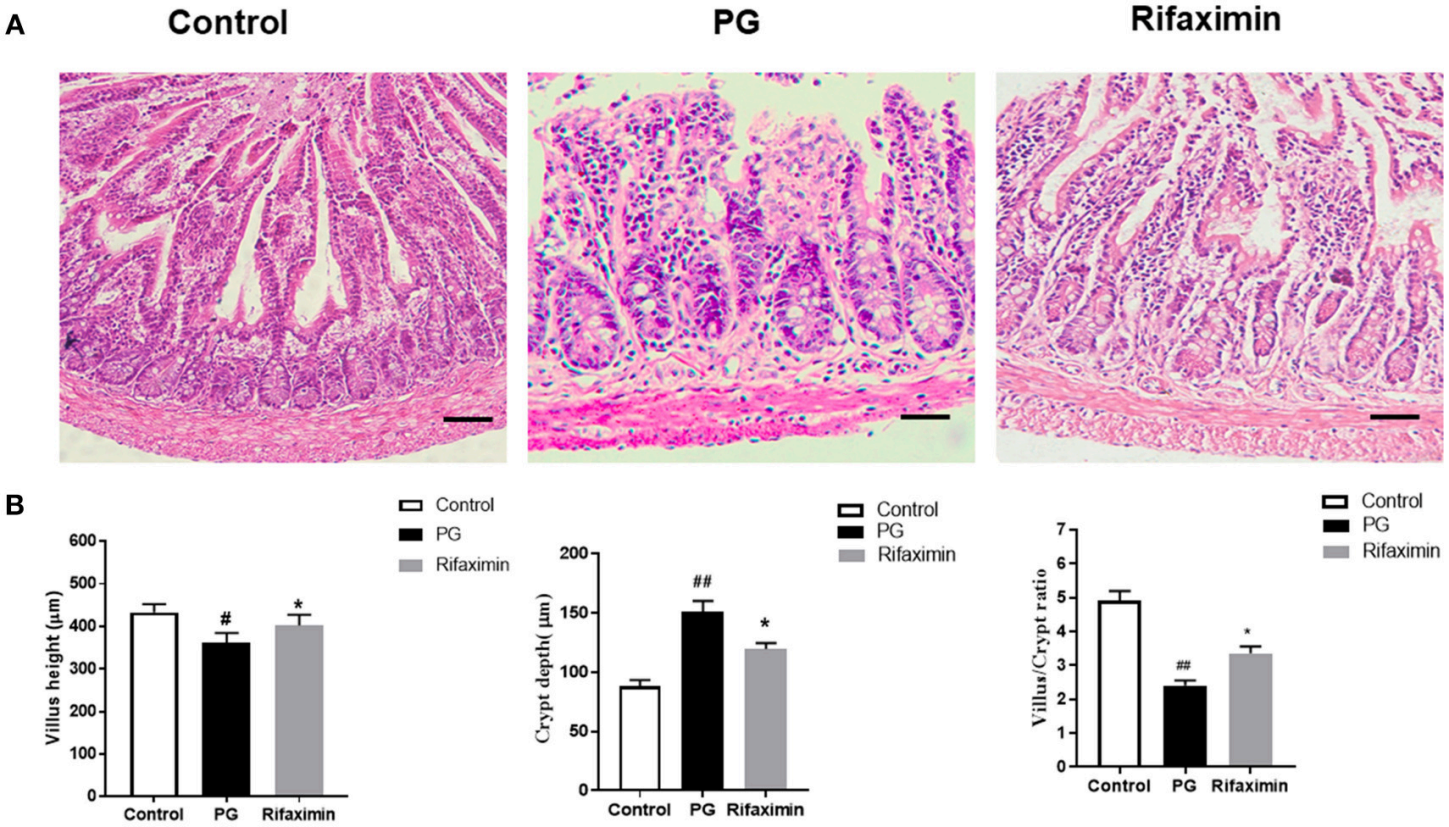
Rifaximin reduces ileum histological alterations in AS mice. **(A)** Ileum excised at week 14 were stained with H&E (Scale bar is 50μm). **(B)** Statistic data of villus height, crypt depth, and villus/crypt ratio. Data are expressed as means ± standard deviation from 6 mice per group (^#^*P* < 0.05 and ^##^*P* < 0.01 vs. control; ^*^P < 0.05 vs. PG alone).

We next examined whether there are intestinal mucosal alterations during the development of AS in pathological sections of the ileum using H&E staining (Figure 3A). Analyses indicated a normal histological morphology in the control group, exhibiting densely packed and intact villi. In contrast with mice in the control or rifaximin groups, PG mice showed increased the crypt depth and decreased the villus height and ratio of villus height to crypt depth (Figures 3A,B). However, administration of rifaximin significantly reduces these pathological alternations.

### Rifaximin Improves the Intestinal Mucosal Barrier Function

**Figure 4:**
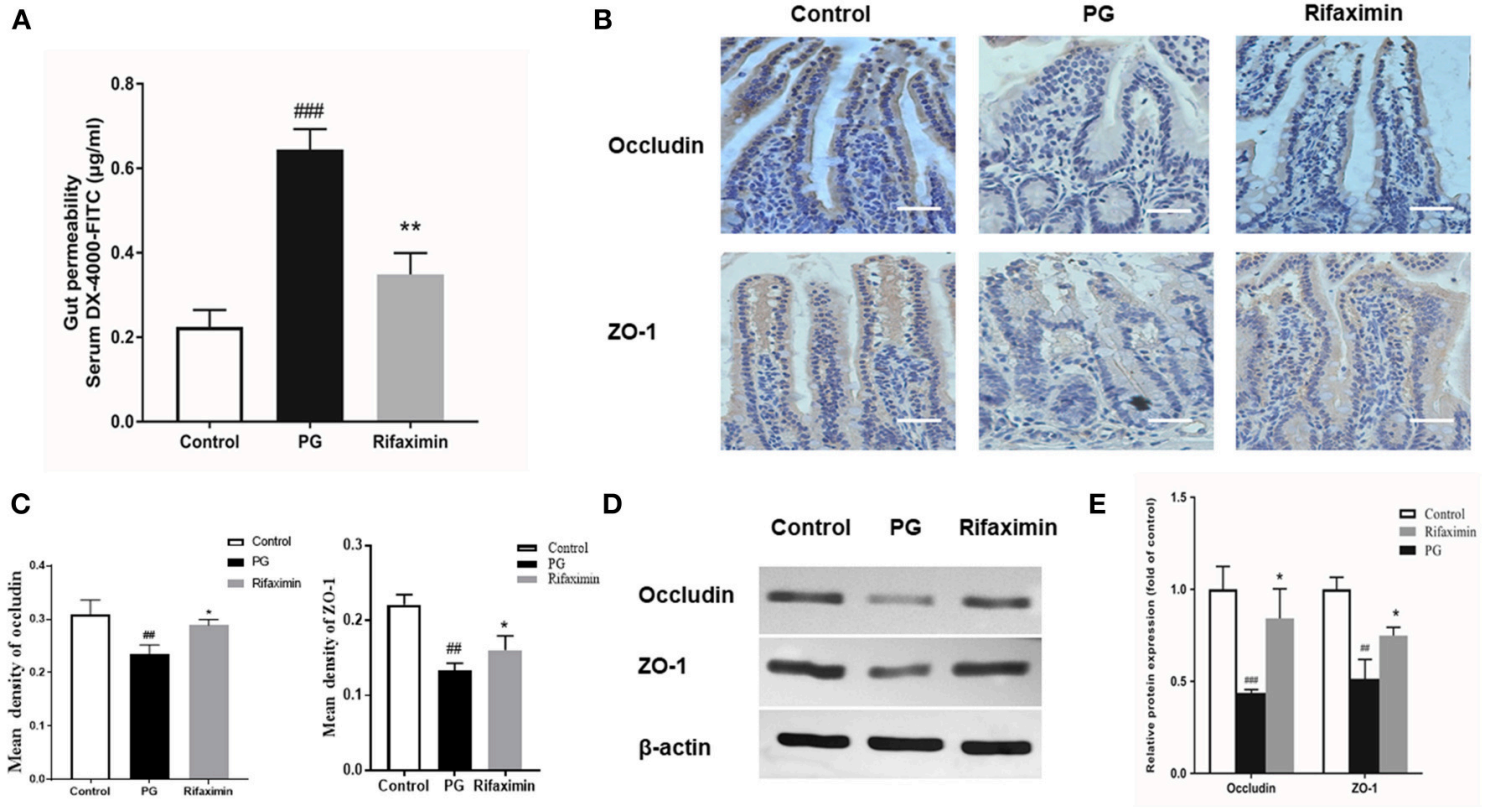
Rifaximin improves the intestinal mucosal barrier function in AS mice. **(A)** Gut permeability was measured by the DX-4000-FITC levels in the serum (*n* = 10 mice per group). **(B)** Occludin and ZO-1 protein in the ileum measured using immunohistochemistry. Occludin and ZO-1 positive cells were stained brown. The more intense the color, the higher the levels of occludin or ZO-1protein (Scale bar is 50μm). **(C)** Representative staining intensities of occludin and ZO-1 as designated by the mean optical density (*n* = 6 mice per group). **(D)** Representative western blots for occludin and ZO-1 proteins expressed in ileal tissue. **(E)** Protein expression quantified using densitometric analysis and was normalized to the levels of β-actin protein (*n* = 10 mice per group). Data are presented as means ± standard deviation (^##^*P* < 0.01, and ^###^*P* < 0.001 vs. control; ^*^P < 0.05, and ^**^P < 0.01 vs. PG alone).

When the epithelium is injured, the concentration of DX-4000-FITC in serum increases so that it can be an indicator of intestinal mucosal integrity. We found that PG induced inflammation required a mechanism involving the control of gut permeability. The PG group had higher body serum DX-4000-FITC levels compared to those in controls, indicating increased intestinal permeability. Rifaximin treatment significantly decreased DX-4000-FITC levels when compared to the PG group (Figure 4A).

Decrease in the expressions of ZO-1 and occludin (key markers of tight junction integrity) can increase intestinal permeability and is closely related with pathophysiology of AS. Immunohistochemistry and western blots were carried out to evaluate the levels of ZO-1 and occludin in the gut. As compared with those of the controls, the protein expression levels of occludin and ZO-1 in the ileum in PG group were significantly decreased (Figures 4B–E). After treatment with rifaximin, the expression of both ZO-1 and occludin in the gut partly recovered when compared to those of the PG group. The results suggest that PG-induced intestinal mucosal barrier dysfunction can be partially protected by rifaximin treatment.

### Rifaximin Inhibits TLR4/NF-kB Pathway Activation in AS Mice

**Figure 5:**
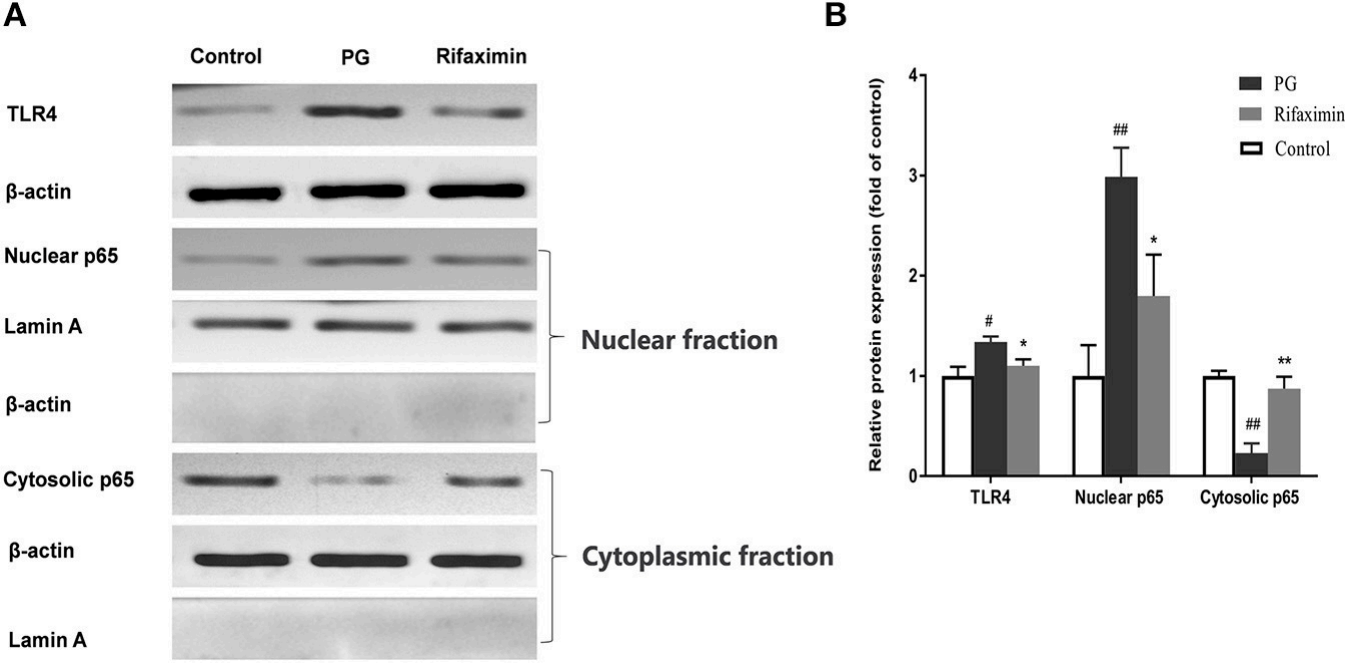
Rifaximin inhibits TLR4/NF-kB pathway activation in AS mice. **(A)** Representative western blots for TLR4, nuclear p65, and cytosolic p65 proteins. **(B)** TLR4 and cytosolic p65 protein expression quantified using densitometric analysis with β-actin as an internal control. Lamin A was used as a loading control for nuclear p65. Data are presented as means ± standard deviation from five mice per group (^#^*P* < 0.05, and ^##^*P* < 0.01 vs. control; ^*^P < 0.05, and ^**^P < 0.01 vs. PG alone).

The interaction between intestinal epithelial cells and microbes is partly mediated by TLR4, meanwhile NF-κB is viewed as a master switch in the regulation of inflammation and immunity. The nuclear translocation of the NF-κB p65 subunit is a key step of NF-κB activation. The existence of NF-κB activation in the ileum of the PG group, was demonstrated by an increase in the translocation of the p65 subunit from the cytosol to the nucleus (Figure 5, *P* < 0·01 vs. normal controls). There was also a significant increase (*P* < 0·01 vs. normal controls) in the protein expression of TLR4 in the PG group. Whereas, rifaximin treated mice displayed both decreased expression of TLR4, and nuclear location of NF-κB p65 protein compared to PG mice. These results indicate that rifaximin can inhibit TLR4/NF-κB activation.

### Rifaximin Modulates the Composition of the Microbiota in AS Mice

**Figure 6:**
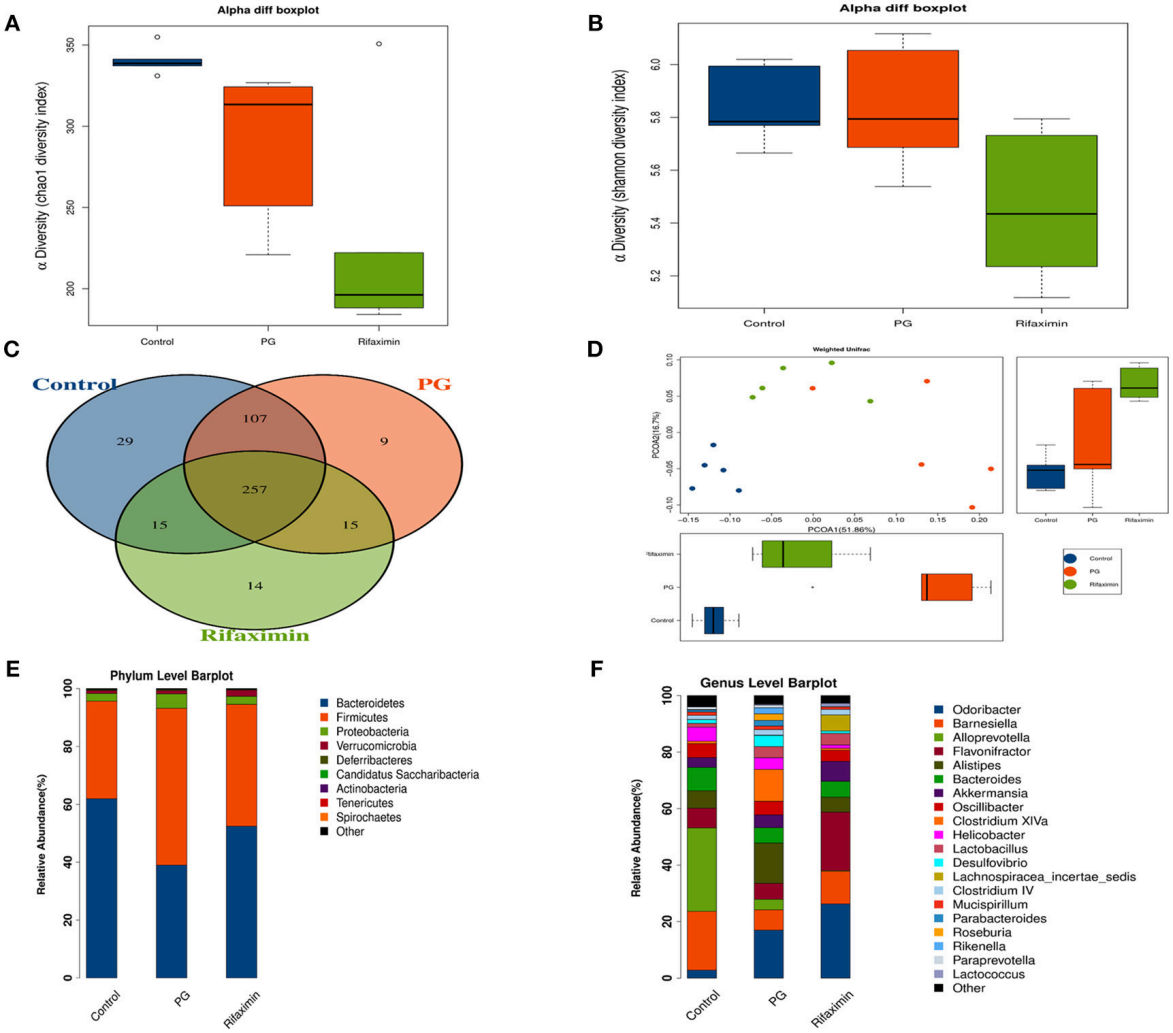
Rifaximin modulates the composition of the microbiota in AS mice. **(A)** Chao1 index and **(B)** Shannon index for the three groups. **(C)** Venn diagram of shared and unique OTUs among different groups. **(D)** Principle coordinate analysis (PCoA) of weighted UniFrac distances. Analysis of the composition of bacteria present at phylum **(E)** and genus levels **(F)**. (*n* = 5 mice per group).

The fecal flora composition of mice from the normal, PG, and rifaximin groups were measured by 16S rDNA sequencing. The alpha diversity was estimated by Chao1 index and Shannon index. The alpha diversity assessed by the number of Chao1 was significantly reduced in the rifaximin group when compared to the control and PG groups (*p* < 0.05, Figure 6A). Shannon diversity showed no significant differences between the three groups, but rifaximin group showed a small decrease in Shannon diversity when compared to the control and PG groups (Figure 6B). The Venn diagrams showed overlapping OTU data for the three groups, which enabled us to distinguish microbes between groups. We found that 257 of 446 OTUs were universal to all of the groups. The rifaximin group had 14 distinct microbes, whereas distinct microbes in the PG and control groups were 9 and 29, respectively (Figure 6C).

In order to evaluate the microbial community structure (β-diversity) between different groups, principal coordinate analysis (PCoA) of weighted UniFrac was performed. PCoA (Figure 6D) showed that the microbial community composition of the three groups were significantly different from each other (Adonis test, *p* < 0.01), suggesting that dysbiosis may be an additional symptom in AS mice.

On the phylum level, *Bacteroidetes* and *Firmicutes* were the two dominant taxa in the three groups (Figure 6E). In the control group, *Bacteroidetes* were the most numerous (61.6%) and *Firmicutes* (33.5%). However, in the PG group there were more *Firmicutes* (53.8%) than *Bacteroidetes* (38.7%). Rifaximin treatment increased the population of *Bacteroidetes* by 52.2% and decreased the proportion of *Firmicutes* by 41.9%. The ratio of *Bacteroidetes/ Firmicutes* was altered between the normal group (1.83) and PG group (0.71). Following rifaximin treatment, the ratio of *Bacteroidetes/ Firmicutes* rose to 1.24. Moreover, PG increased the microbial proportion of the phyla *Proteobacteria*, which is different to that seen in the other two groups.

**Figure 7:**
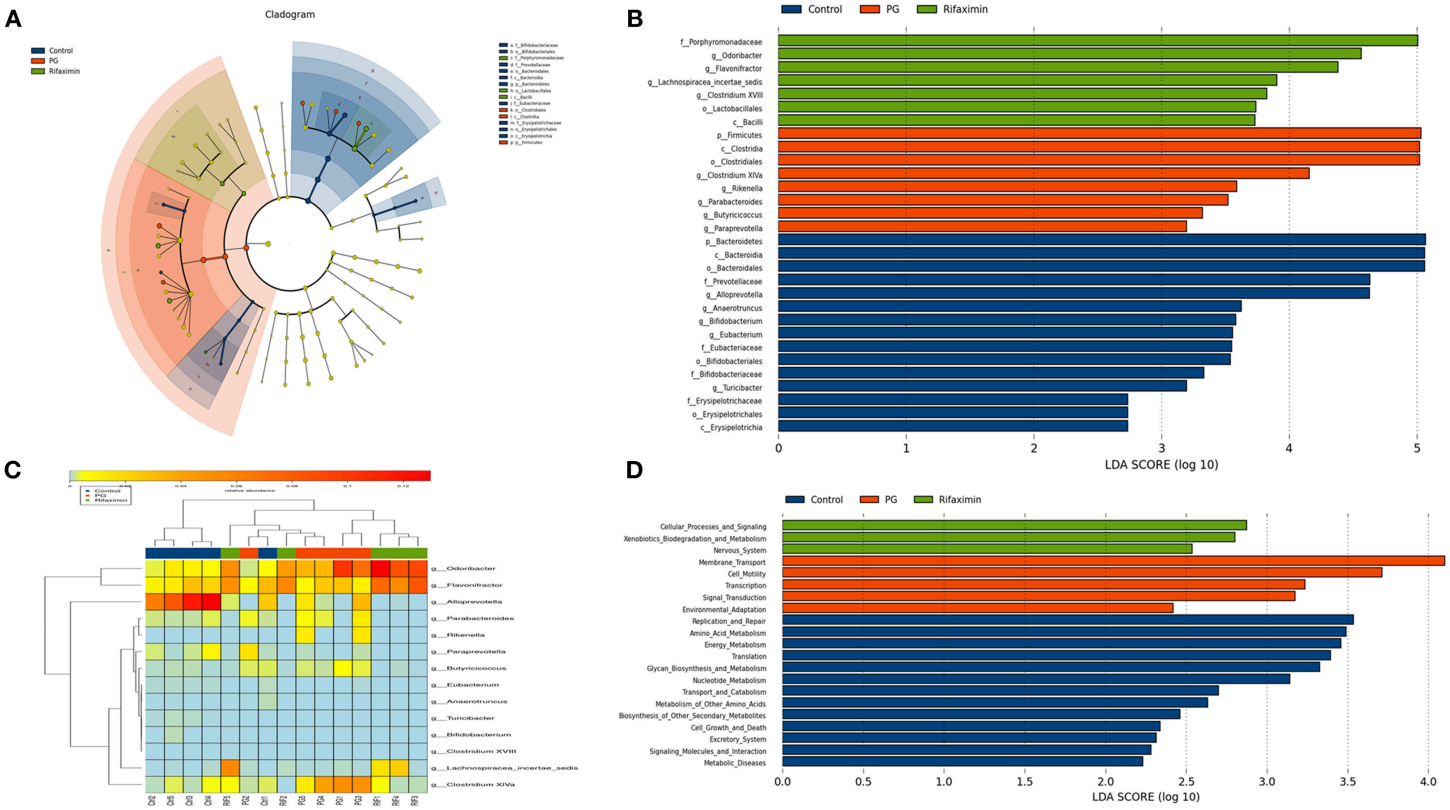
Comparison of the gut bacterial taxa and functional differences between treatment groups. **(A)** Cladogram generated by LEfSe analysis of gut microbiota in the different groups. **(B)** Bacterial taxa differences among the three groups were detected by LefSe (*p* < 0.05, linear discriminant analysis >2 logs). **(C)** Heat map of the 14 most abundant taxons in the three groups, at the genus level. **(D)** Distribution of Kyoto Encyclopedia of Genes and Gemos (KEGG) functional categories of KEGG Ortholog (KO) markers. Comparison among three group markers on level 2 of the KEGG functional category.

On the genus level, we have found that the gut microbial community of the three groups are different (Figure 6F). LEfSe analysis was used to determine the key alteration of fecal flora in mice (*P* < 0.05, LDA score > 2.0). We found that eight bacterial taxa were significantly higher in the PG group, and seven taxa (including *Lactobacillales*) were enriched in the rifaximin treatment group (Figures 7A,B). We generated a heat map showing 14 abundant taxons which were significantly different between the groups at the genus level (Figure 7C, Kruskal-Wallis, all pairwise comparisons, *P* < 0.05). Contrasted with control group, the relative abundance of *Odoribacter, Clostridium XlVa, Parabacteroides, Butyricicoccus*, and *Rikenella* in the PG group were all higher, whereas the relative abundance of *Alloprevotella, Eubacterium, Anaerotruncus, Turicibacter*, and *Bifidobacterium* were lower (*P* < 0.05). Rifaximin treatment significantly decreased the abundance of *Rikenella, Clostridium XlVa*, and *Butyricicoccus* when compared to the PG group (*P* < 0.05). Meanwhile, the abundance of *Flavonifractor*, and *Lachnospiracea_incertae_sedis* in rifaximin group were higher than the other 2 groups (*P* < 0.05).

The PICRUSt programme was used to analyze the functional differences between the three groups. Based on LEfSe, the abundance of three pathways: cellular processes and signaling, xenobiotics biodegradation and metabolism, and nervous system, were elevated in the rifaximin group. Conversely, the KEGG pathways membrane transport, transcription, cell motility, signal transduction, and environmental adaptation were predicted at higher levels in the microbiota from the PG group (Figure 7D).

## Discussion

Although so far the cause of AS is still remaining unclear, numerous studies indicate that AS is closely associated with alterations in the community of the gut microbiota (Costello et al., 2014; Wen et al., 2017). Therefore, the possibility of preventing AS progression by regulating the intestinal flora through antibiotics, prebiotics, or probiotics seems logical. Notably, recent studies have shown that treatment of HLA-B27 rats with oral antibiotics not only resolves their microbiota-dependent intestinal inflammation, but also reduces inflammatory factors in the blood (Ansalone et al., 2017). Rifaximin is a poorly absorbed antibiotic with broad-spectrum activity and previous studies have shown that rifaximin can inhibit inflammatory reaction through modulating gut microbiota (Ponziani et al., 2017). However, the protective effects and mechanism of rifaximin on AS have not been studied yet. This study investigated the effects of rifaximin on PG-induced AS mice. Our results demonstrated that the PG group displayed significantly higher disease indicator arthritis scores and vertebral joint destruction. Meanwhile, rifaximin could significantly relieve the inflammatory response and joint destruction caused by PG. This suggests that rifaximin can delay progression and reduced the severity of AS.

AS is characterized by persistent inflammation, and previous research has shown that elevated circulating levels of inflammatory factors such as IL-17, TNF-α, IL-6, and IL-23 are important in the pathogenesis of AS. Blocking these inflammatory factors with TNF-alpha blocking agents and other biological agents, could reduce peripheral and axial joints inflammation in AS (Chen et al., 2015). Brown et al. (2009) found that pretreatment of epithelial cells with rifaximin altered bacterial attachment and reduced the concentrations of pro-inflammatory cytokines, such as IL-6, IL-8, and IL-15 *in vitro*. Jin et al. (2017) found that rifaximin could inhibit the expression of IL-12 and IL-17 in ileum of irritable bowel syndrome mice. In addition, our data indicated that rifaximin significantly reduced the secretion of TNF-α, IL-6, IL-17A, and IL-23 in AS mice. IL-23/IL-17 signaling pathways has been known to trigger chronic inflammation in AS. Previous research has shown that microbial antigens may result in increased expression of inflammatory factors including IL-23 and IL-17 (Rosenbaum and Asquith, 2016). For example, *Salmonella typhimurium* can stimulate synovial fluid mononuclear cells to release IL-17 and IL-23 in patients suffer from reactive arthritis and undifferentiated spondyloarthropathy (Chaurasia et al., 2016). Our results demonstrated that rifaximin could inhibit the development of AS in mice by reducing the production of pro-inflammatory cytokines.

The intestinal epithelial barrier plays an important role in prohibiting translocation of bacteria, while barrier disruption can increase intestinal permeability, which may provoke immune responses in various diseases (Andersen et al., 2017; Lin and Zhang, 2017). There are ~60% of patients with SpA who also suffer from subclinical gut inflammation, ~10% of those with obvious gut inflammation evolving into Crohn's disease (Ciccia et al., 2016). Previously, it has been reported that patients with AS and their first-degree relatives have increased gut permeability (Martinez-Gonzalez et al., 1994). In addition, Kerr et al. demonstrated that HLA-B27 rats had nearly five times level of gut permeability than healthy control rats (Kerr et al., 1999). Tight junction (TJ) proteins suppress paracellular permeability and therefore are the main determining factors in gut barrier function. Previous research found that chronic stress-induced impairment of intestinal barrier function was attenuated by the rifaximin through upregulating the expression of occludin (Xu et al., 2014). Jin et al. (2017) also found that rifaximin could reduce intestinal permeability and promoted the expression of the major TJ protein occluding in mice with irritable bowel syndrome. In this research, our data shown that occludin and ZO-1 were significantly depressed in AS mice. Notably, rifaximin could protect the intestinal mucosal barrier effectively by increasing the expression of TJ proteins, including ZO-1 and occludin. At the same time, the levels of serum DX-4000-FITC were decreased after rifaximin treatment. Moreover, we found administration of rifaximin significantly increased the villus height and ratio of villus height to crypt depth in ileum. Increasing the villus height indicates that enhancing the efficiency of digestion and absorption, thus long villi are correlated with improved gut health (Laudadio et al., 2012).

The mechanism of action of rifaximin on intestinal barrier integrity and anti-inflammatory properties is currently unknown. We investigated a potential beneficial role for rifaximin through the regulation of intestinal microbes. Previous research has found that the ratio of *Bacteroidetes*/*Firmicutes* can be used to evaluate whether intestinal flora is balance or not (Murphy et al., 2010). Patients with SpA have been found to have microbial dysbiosis with a decreased *Bacteroidetes*/*Firmicutes* ratio (Breban et al., 2017). In this study, levels of *Bacteroides* in the PG group was significantly decreased compared with control group, but level of *Firmicutes* was significantly increased. However, the trend was reversed by rifaximin. Under certain circumstances, increased *Firmicutes* and decrease *Bacteroidetes* levels can induce an immune response and activation of Thl7 cells (Magrone and Jirillo, 2013). Kim et al. (2012), found that a high fat diet induced macrophage infiltration and inflammation in an obese mouse model, which was associated with intestinal flora dysbiosis by increasing the ratio of *Firmicutes*/ *Bacteriodetes*. In addition, some authors have reported that flora dysbiosis and TLR-4/NF-κB pathway activation were related to inflammasome initiation response (Porras et al., 2017). Studies have shown that rifaximin can suppress microbes-induced immune response through inhibiting the activation of NF-κB via the pregnane X receptor(Jin et al., 2017; Lopetuso et al., 2018). In our study, we focused on the TLR-4 which has been shown to play a key role in the many inflammatory diseases. Dysbiosis of gut microbiota can lead to increase endotoxin like lipopolysaccharide (LPS) into the body and the binding of LPS to TLR-4 can induce NF-κB activation(Gil-Cardoso et al., 2016). Our study showed that intestinal mucosal barrier damage in AS mice was related to TLR-4-mediated NF-κB activation, and then promoted the release of inflammatory factors. Rifaximin treatment could attenuate TLR-4/NF-κB pathway activation in the gut.

The gut microbial community of the three groups is also remarkably different on the genus level. Previous studies have demonstrated that the *Rikenellaceae* family is the most abundant in AS patients (Bazin et al., 2018). In our study, the abundance of *Rikenella* (belonging to the *Rikenellaceae* family) increased greatly in the PG group, but decreased following rifaximin treatment. Furthermore, it has been reported that rifaximin can lead to a relative abundance of beneficial intestinal bacteria, such as *Lactobacillus* and *Bifidobacterium* (Jin et al., 2017). Xu et al. indicated that rifaximin can stimulate the growth of *Lactobacilli* which can prevent chronic stress induced-mucosal inflammation, impairment of the intestinal barrier function, and visceral hyperalgesia (Xu et al., 2014). They also found that the increased population of *Lactobaccillus* was related to the decrease of diversity after rifaximin treatment. In this study, we also found that rifaximin treatment produced a higher population of *Lactobacillus tender* in feces. *Lactobacilli* have been reported to down-regulate pro-inflammatory factors, modulate intestinal permeability and prevent the translocation of pathogenic bacteria (Bäuerl et al., 2013). But there were no significant differences between the three groups in the abundance of *Bifidobacterium* in our study. Some research shows that when comparing *in vivo* mouse data derived from different research institutions, intestinal microbiota composition could be differences (Thoene-Reineke et al., 2014). Actually, not only genetic background, infection status and intervention but also housing condition can modulate the intestinal microbiota composition. Even mice from the same provider and given the same diet, intestinal microbiota composition in mice vary between housing conditions (Xiao et al., 2015).

In this study, the fecal metagenomes were predicted using PICRUSt (Langille et al., 2013), and we identified several different KEGG pathways as differentially expressed amongst the three groups. Increased abundance of genes associated with membrane transport were found in AS mice, similar to data observed in patients suffer from IBD and obesity (Greenblum et al., 2012). A further important pathway altered in mice with AS was the metabolism of cell motility. Michelle et al. found that gut microbiomes with active colitis were enriched in groups associated with cell motility including genes for flagellar assembly (Rooks et al., 2014). In the mouse model of colitis and human IBD, bacterial flagella antigens have been identified as the driving factor (Rosser et al., 2014). In contrast, rifaximin-enriched genes were frequently involved in cellular processes and signaling, xenobiotics, biodegradation, and metabolism. Xenobiotic detoxification systems are able to remove compounds from the complex mixtures produced by metabolic process or the environment (Cheng et al., 2013). The human intestinal flora makes an important contribution to the metabolism of xenobiotics. The intestinal flora can change the chemical structure of ingested substances and then can affect xenobiotic toxicity, biological activity, and bioavailability, thereby regulating host metabolism which promote many xenobiotics removed from the body (Koppel et al., 2017). Therefore, rifaximin may promote the degradation of xenobiotics in AS mice by regulating gut flora. However, the effects of rifaximin on cecal microbes and function remain unclear, and its mechanism of action requires further investigation.

**Figure 8:**
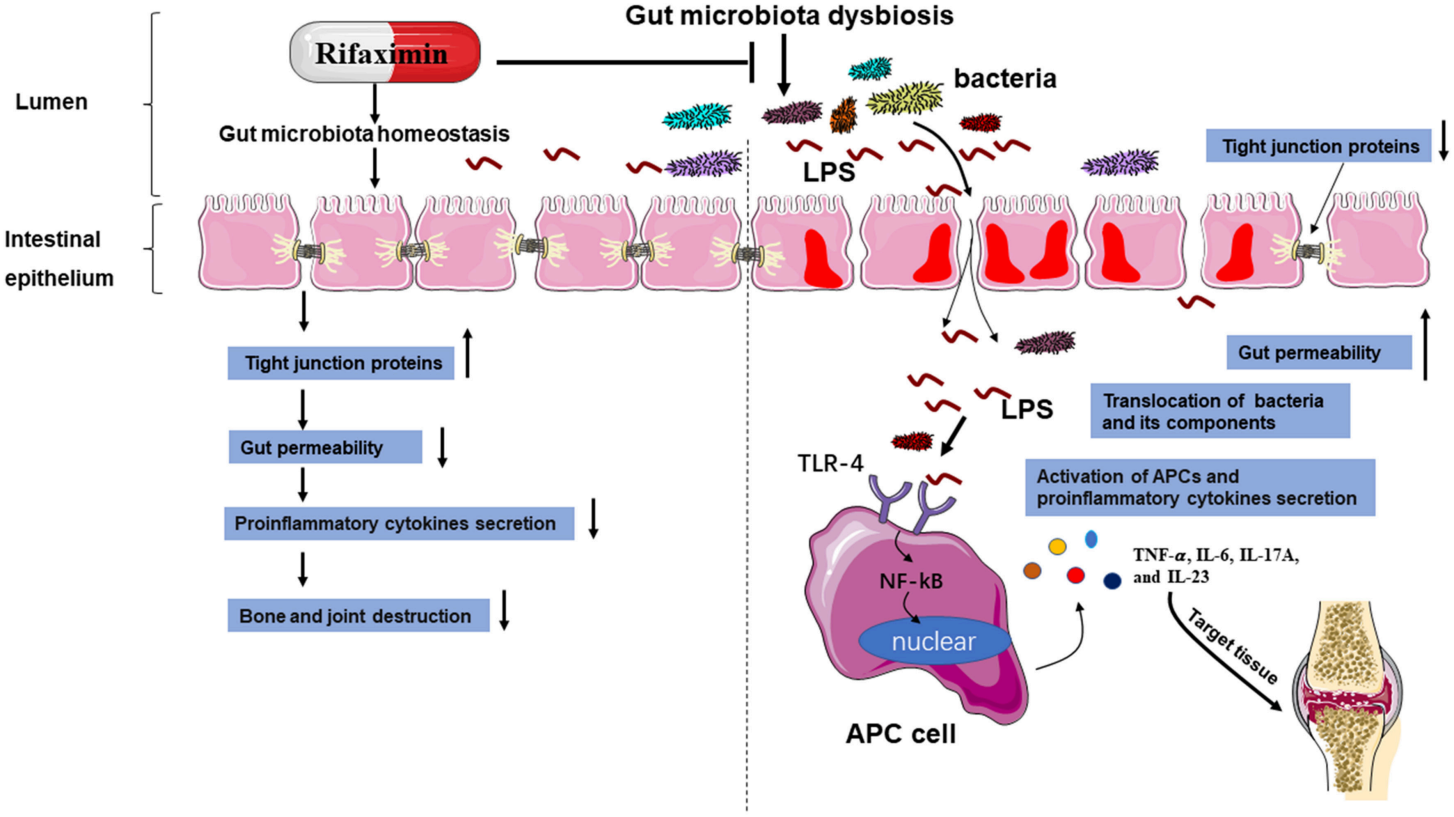
A schematic diagram of the proposed mechanisms of rifaximin on AS. Abbreviations: antigen-presenting cell (APC); toll-like receptor 4 (TLR-4); lipopolysaccharide (LPS).

In conclusion, the present study showed that rifaximin might be a new promising option to treat AS, and highlights the regulation of intestinal microbiota as a promising therapy strategy for the treatment of AS. We have demonstrated that rifaximin inhibits PG-induced AS development by working as an anti-inflammatory prebiotic, reducing pathobionts from the intestinal microbiota, and has a positive effect on the intestinal environment. Its mechanism of action is in part through downregulation of the TLR4/ NF-κB pathway activation and regulating gut microbiota (Figure 8). However, more detailed research is required to establish the precise molecular mechanisms involved. This may ultimately lead to the development of a new therapy for the inhibition of AS.

## Author Contributions

LY, BL, and JZ carried out the study and wrote the manuscript. JH, JL, ZS, and QZ collected samples. MW, ZC, and TW analyzed data. WZ and QL carried out literature search, and manuscript editing. HL designed the study and revised the manuscript.

### Conflict of Interest Statement

The authors declare that the research was conducted in the absence of any commercial or financial relationships that could be construed as a potential conflict of interest.
